# Supplementary material for: The EMC acts as a chaperone for membrane proteins
Source: Nat Commun. 2025 Aug 2;16:7097. doi: 10.1038/s41467-025-62109-x (PMC12317982; doi:10.1038/s41467-025-62109-x)
Supplement: Supplementary file 1 — Supplementary Information [file 41467_2025_62109_MOESM1_ESM.pdf]

The EMC acts as a chaperone for membrane proteins

Carolin J. Klose[1,2,a], Kevin Meighen-Berger[1,a], Martin Kulke[1], Marina Parr[3], Barbara Steigenberger[4], Martin Zacharias[1], Dmitrij Frishman[3], and Matthias J. Feige[1,*]

[1]Center for Functional Protein Assemblies (CPA), Department of Bioscience, TUM School of Natural Sciences, Technical University of Munich, 85748 Garching, Germany

[2]Department of Molecular Machines and Signaling, Max Planck Institute of Biochemistry, 82152 Martinsried, Germany

[3]Department of Bioinformatics, Wissenschaftszentrum Weihenstephan, Technical University of Munich, 85354 Freising, Germany

[4]Mass Spectrometry Core Facility, Max Planck Institute of Biochemistry, 82152 Martinsried, Germany

[a]these authors contributed equally

[*]Corresponding author, email: Matthias.Feige@tum.de

Supplementary Fig. 1

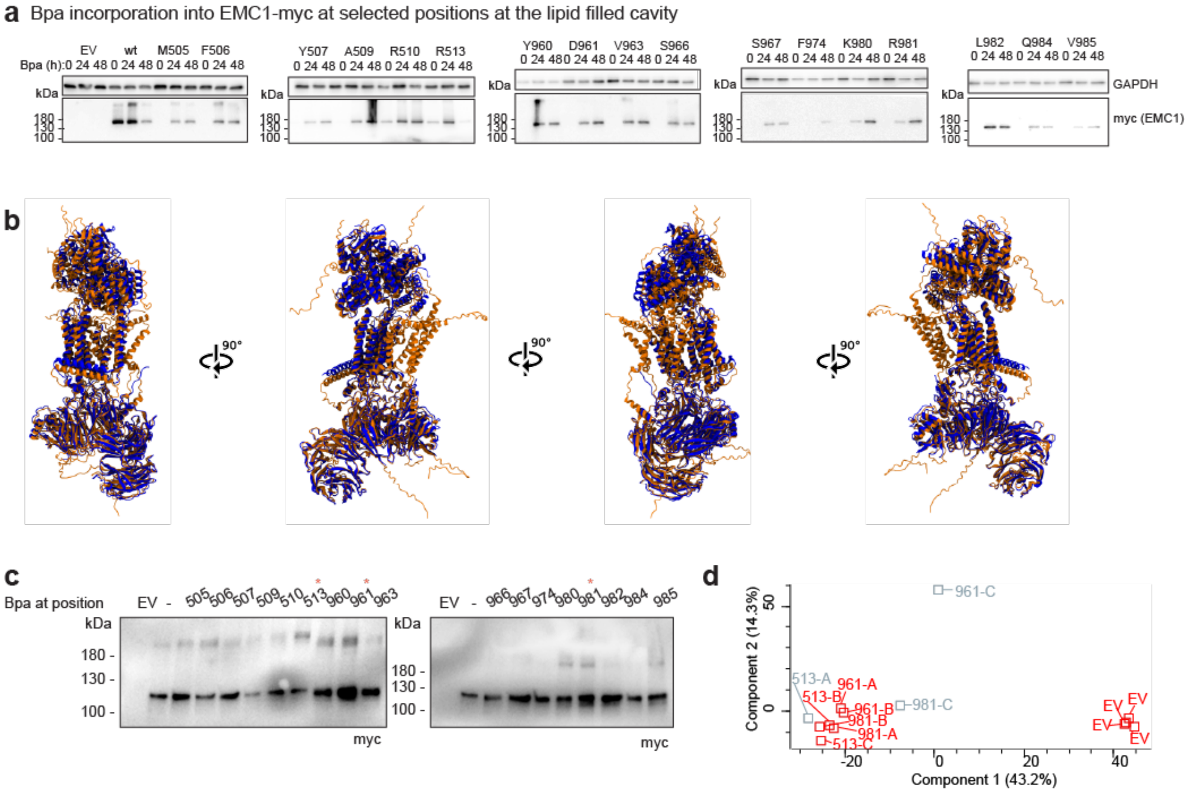

Supplementary Figure 1. Incorporation of Bpa at the EMC lipid-filled cavity by genetic code expansion for mass spectrometry.

a, Site-specific incorporation of Bpa into EMC1-myc by genetic code expansion in HEK293T cells at 17 different positions lining the lipid-filled cavity. Western blots of whole cell lysates against the C-terminal myc epitope tag of EMC1 allows the evaluation of the efficiency of amber codon suppression. Comparison of no addition of Bpa (-) and two different expression durations (24 h and 48 h) revealed no significant differences for most positions, hence 24 h were chosen for all subsequent experiments.

b, Different orientations of the EMC Alphafold 2 multimer model (in orange) fitted onto an experimentally determined EMC structure in blue (PDB code 8EOI). This Alphafold 2 multimer model, also containing parts of the EMC that have not been resolved in experimental structures, was used throughout this study.

c, Evaluation of the UV-induced crosslinking efficiency at selected EMC1 positions to endogenous interaction partners. Bpa was incorporated into EMC1 at indicated positions, in-cell crosslinking was induced by UV irradiation prior to cell lysis and EMC1-myc and covalent adducts were immunoprecipitated using the myc epitope tag and analyzed by western blot. Positions chosen for further analysis by mass spectrometry are marked with a red asterisk.

d, Principal component analysis (PCA) plot of all replicates measured by mass spectrometry. Lables are according to the position replaced by Bpa (981, 961, 513) or empty vector (EV). Datasets labled in grey were excluded from the final analysis.

Supplementary Fig. 2

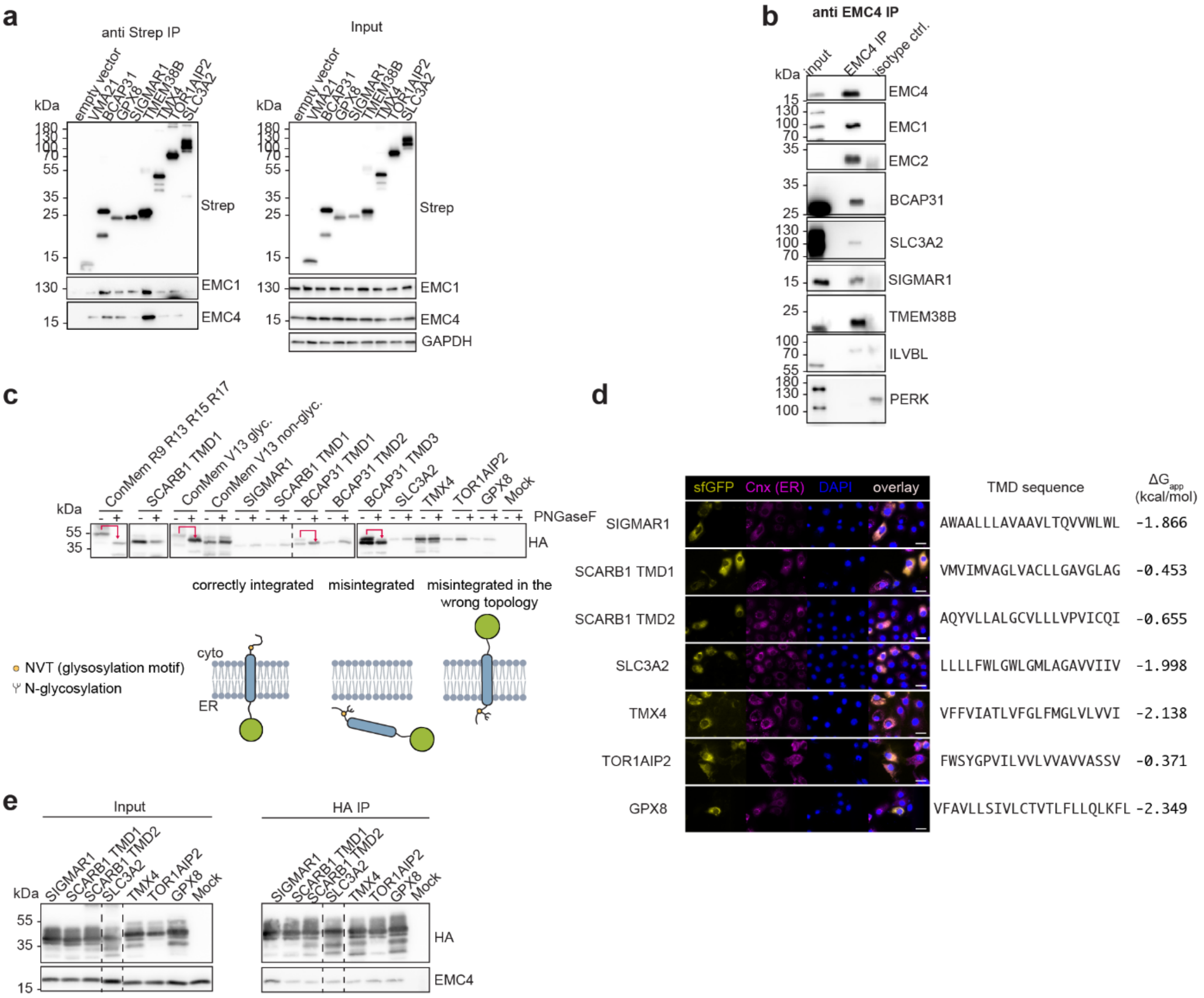

Supplementary Figure 2. Validation of selected EMC1 interactors.

a, Validation of selected EMC interactors identified in Fig. 1c. Potentially interacting proteins bearing a cytoplasmic twinStrep tag were transiently expressed in HEK 293T cells and in-cell crosslinking with DSP was carried out prior to cell lysis and immunoprecipitation. Interaction with the EMC was validated by western blot against EMC1 and EMC4.

b, The interaction was further assessed at endogenous levels for a subset of proteins. Endogenous EMC4 was isolated via immunoprecipitation and interaction with other proteins was assessed by western blot. PERK and ILVBL served as negative controls to exclude unspecific intramembrane binding.

c, PNGaseF digest of the model proteins described in Fig. 1d to assess their membrane integration. Top: Constructs containing a cytoplasmic glycosylation site, meaning that shifts indicate full transport of the constructs into the ER lumen. ConMem containing only a cytoplasmic glycosylation site (V13 non-glyc.) and ConMem containing only an ER lumenal glycosylation site (V13 glyc.) were used as negative and positive controls, respectively, and are described in more detail in Fig. 2a and in.[1] Furthermore, a ConMem variant that fails to

integrate into the membrane due to its multitude arginine residues was included as a positive control for glycosylation upon full ER translocation. Bottom: Schematics depicting the principle of the reporter constructs. If the reporters are correctly integrated into the membrane (left), their glycosylation site is not exposed to the ER and thus they will remain unglycosylated, which can then be verified by PNGaseF digest. If they are misintegrated (middle), they are N-glycosylated and thus size-shifts can be oserved via western blot upon PNGaseF digest. Alternatively, if they were misintegrated in the wrong topology (right), they would also be N-glycosylated and thus size-shifts would be observed via western blot upon PNGaseF digest. In these assays, BCAP TMD1 and TMD3 were found to be misintegrated and were thus omitted in further experiments.

d, Fluorescence microscopy of COS-7 cells expressing the model proteins described in Fig. 1d using a DMi8 widefield fluorescence microscope. Superfolder (sf) GFP signal is depicted in yellow, the ER is stained *via* anti-calnexin antibody (in magenta), and nuclei are stained with DAPI (in blue). The isolated transmembrane domain sequences of the respective proteins are shown in addition to predicted ΔG_{app} values for insertion of these regions into the ER membrane by means of the Sec61 translocon, as calculated with DGPred.[2]

e, Representative western blots used for quantification of the EMC4:model protein interaction *via* co-immunoprecipitation depicted in Fig. 1d. The whole cell lysate (input) and HA pulldowns were blotted. Non-relevant lanes were cropped, as indicated by dashed lines.

Supplementary Fig. 3

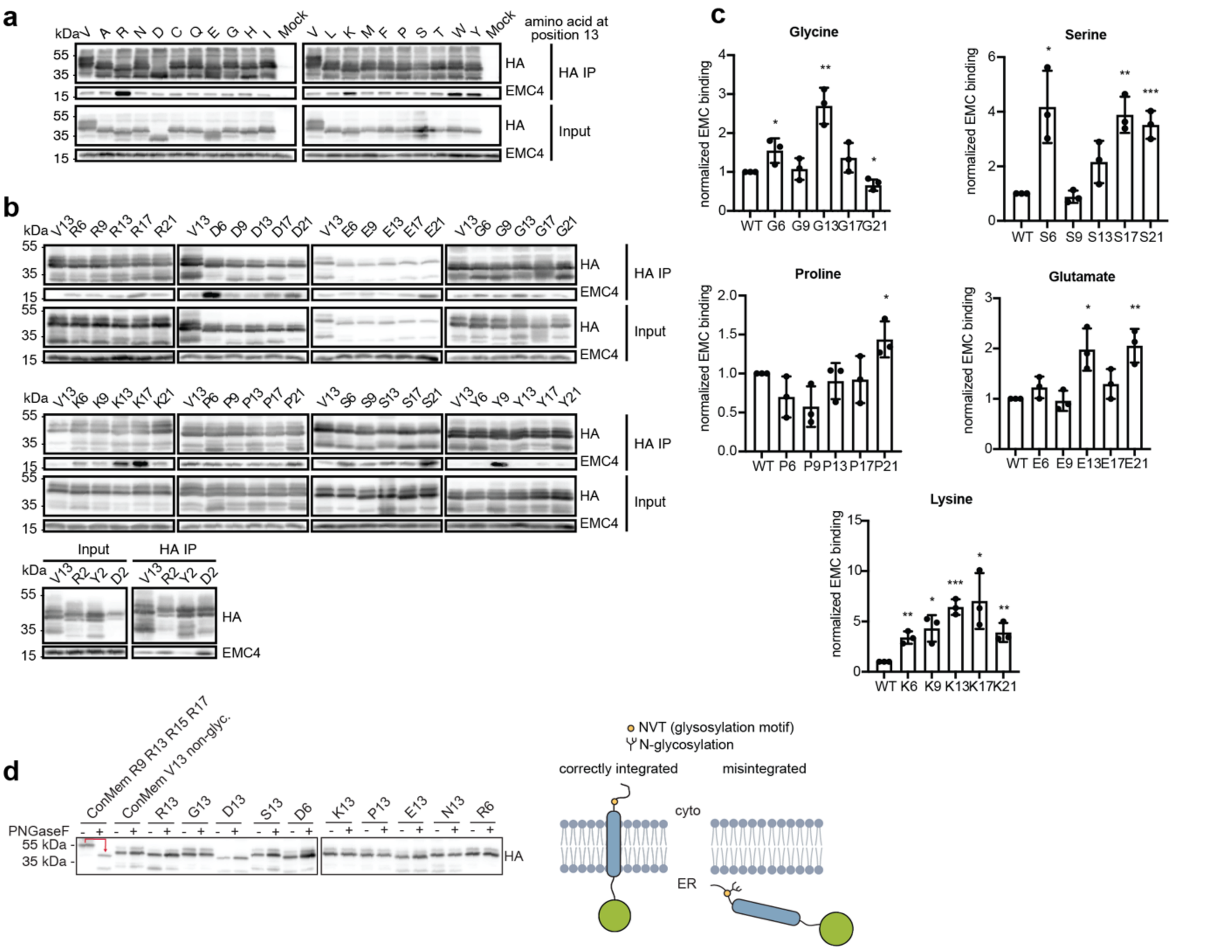

Supplementary Figure 3. Assessing the interaction of the EMC with ConMem variants.

a, Representative western blots of the interaction of EMC4 with different position-13 ConMem variants assessed *via* co-immunoprecipitation, of which the quantification is shown in Fig. 2b. The whole cell lysate (input) and HA pulldowns were blotted.

b, Representative western blots depicting the interaction of EMC4 with ConMem variants bearing amino acid substitutions shifted throughout the membrane, assessed *via* co-immunoprecipitation, of which the quantification is shown in Fig. 2c and Extended Data Fig. 3c. The whole cell lysate (input) and HA pulldowns were blotted.

c, Profiling positional binding dependency of the EMC to ConMem variants with substitutions at indicated positions throughout the TMD. Three amino acid profiles are shown in Fig 2c, the remaining five of the assessed eight substituted amino acid profiles are shown here. Binding was assessed by co-IP in three independent replicates and normalized to the original consensus sequence (mean ± SD, *P value < 0.05, **P value < 0.01, ***P value < 0.001, two-tailed Student's t tests).

d, PNGaseF digest of a select group of ConMem variants. A ConMem variants fully translocated into the ER lumen was included as a positive control and a ConMem variant with

no ER-exposed glycosylation sites was included as a negative control. Right: A schematic depicting the principle of this assay, as explained for Supplementary Fig. 2d.

Supplementary Fig. 4

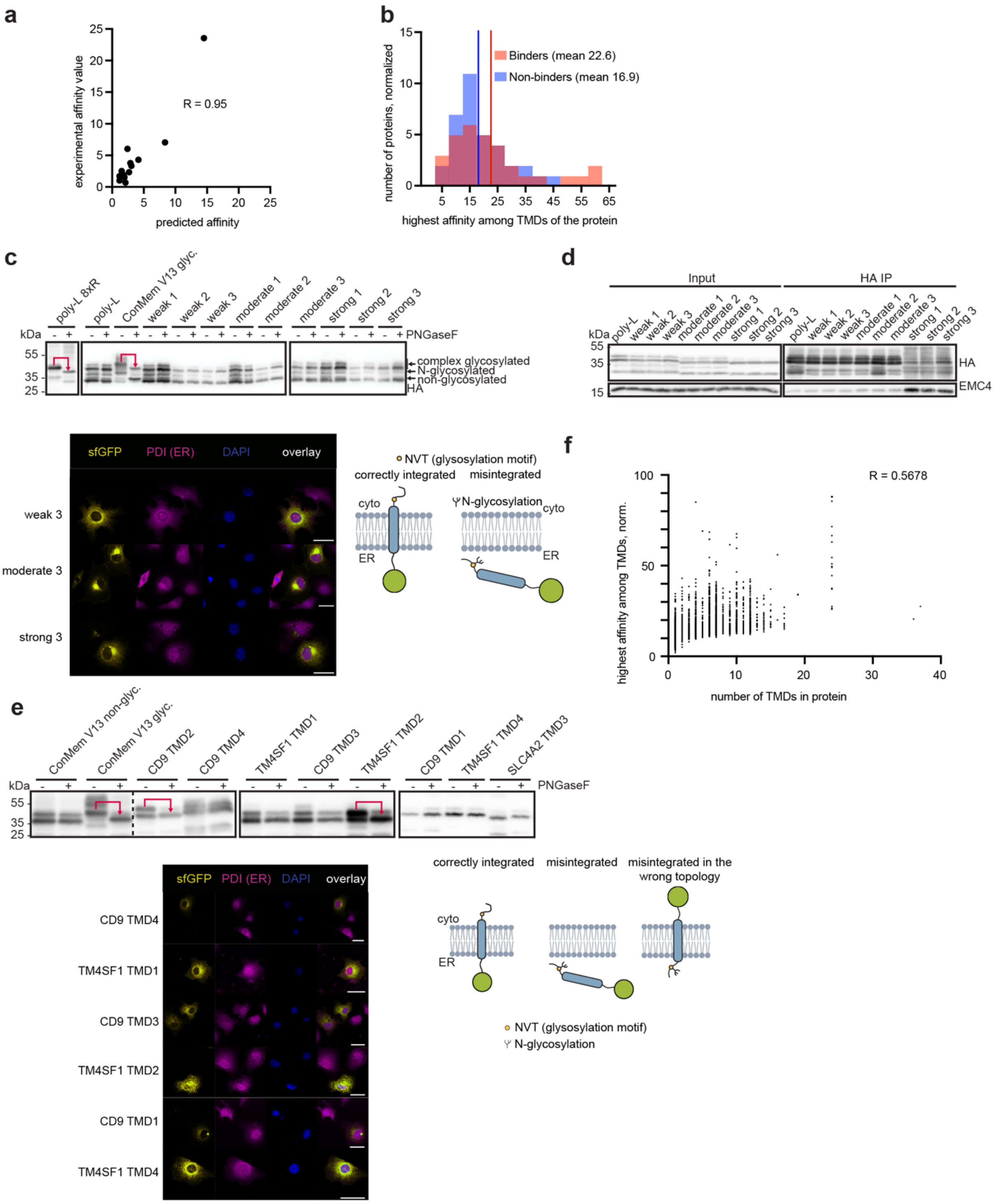

Supplementary Figure 4. Applying ipred^{EMC} to define EMC binding preferences.

a, Correlation between predicted EMC binding affinity and experimentally determined values for a test set of 15 ConMem sequences that were not used for training. Correlation calculated as Pearson correlation coefficient R; R^2 = 0.9025.

b, Verification of the ipred^{EMC} using the mass spectrometry dataset from Figure 1c. The affinity of all TMDs in selected proteins was assessed and the highest affinity was chosen as a

representative value. Binders in the MS dataset on average contained TMDs with a higher affinity than non-binders (22.6 vs 16.9) with a p value of 0.05 (two-sided t-test). Soluble proteins, mitochondrial proteins, EMC subunits and single-pass membrane proteins were exluded from this data.

c, Assessment of the membrane integration of the poly-L constructs described in Fig. 3b. Bottom right: Schematic depicting the two potential scenarios for this type of glycosylation reporter constructs. If the reporters are correctly integrated into the mebrane (left), their glycosylation site is exposed to the cytoplasm and thus will not become N-glycosylated. If they are misintegrated (right), they are N-glycosylated and thus size-shifts can be oserved via western blot upon PNGaseF digest. Top: PNGaseF digest of the poly-L constructs described in Fig. 3b. As these constructs contain only a cytoplasmic glycosylation site if correctly integrated into membrane, absence of shifts upon PNGase F treatment indicates integration of the constructs into the ER membrane in a correct orientation. ConMem with its first, lumenal glycosylation site intact (V13 glyc.) and a variant of the construct containing eight arginines in its TMD (poly-L 8×R) were used as a positive controls. Red arrows indicate observed band shifts upon PNGaseF digest. Bottom left: Fluorescence microscopy of COS-7 cells expressing the model proteins described in Fig. 3b using a STELLARIS 5 confocal microscope platform. Superfolder (sf) GFP signal is depicted in yellow, the ER is stained *via* anti-PDI antibody (in magenta), and nuclei are stained with DAPI (in blue). The observed GFP signal from the constructs overlaps with the ER signal, demonstrating ER localization. Three representative experiments are shown as examples.

d, Representative western blots depicting the interaction of EMC4 with the poly-L constructs, assessed *via* co-immunoprecipitation, of which the quantification is shown in Fig. 3b. The whole cell lysate (input) and HA pulldowns were blotted.

e, Assessment of the membrane integration of the isolated client TMDs described in Fig. 3c. Top: PNGaseF digest of the ConMem-derived constructs containing isolated EMC client TMDs which were predicted to have varying degrees of EMC binding affinity. As these constructs contain only a cytoplasmic glycosylation site, a lack of band shifts indicates integration of the constructs into the ER membrane, as depicted in the schematic. ConMem with its first, lumenal glycosylation site intact was used as a positive control. Bottom: Fluorescence microscopy of COS-7 cells expressing the model proteins described in Fig. 3c using a STELLARIS 5 confocal microscope platform. Superfolder (sf) GFP signal is depicted in yellow, the ER is stained *via* anti-PDI antibody (in magenta), and nuclei are stained with DAPI (in blue). The observed GFP signal from the constructs overlaps with the ER signal, demonstrating ER localization. CD9 TMD2 and TM4SF1 TMD2 were omitted from subsequent experiments due to their misintegration in these assays.

f, Application of the predictor reveals a preference of EMC to bind proteins with more TMDs. The highest affinity among all TMDs of each protein was mapped and this affinity was compared between proteins containing varying numbers of TMDs. The number of proteins was normalized to the total number of proteins in the dataset, and the affinity values, ranging from 0-180, were normalized to 0-100. Correlation calculated as Pearson correlation coefficient R. R^2=0.3223. P (two-tailed)<0.0001.

Supplementary Fig. 5

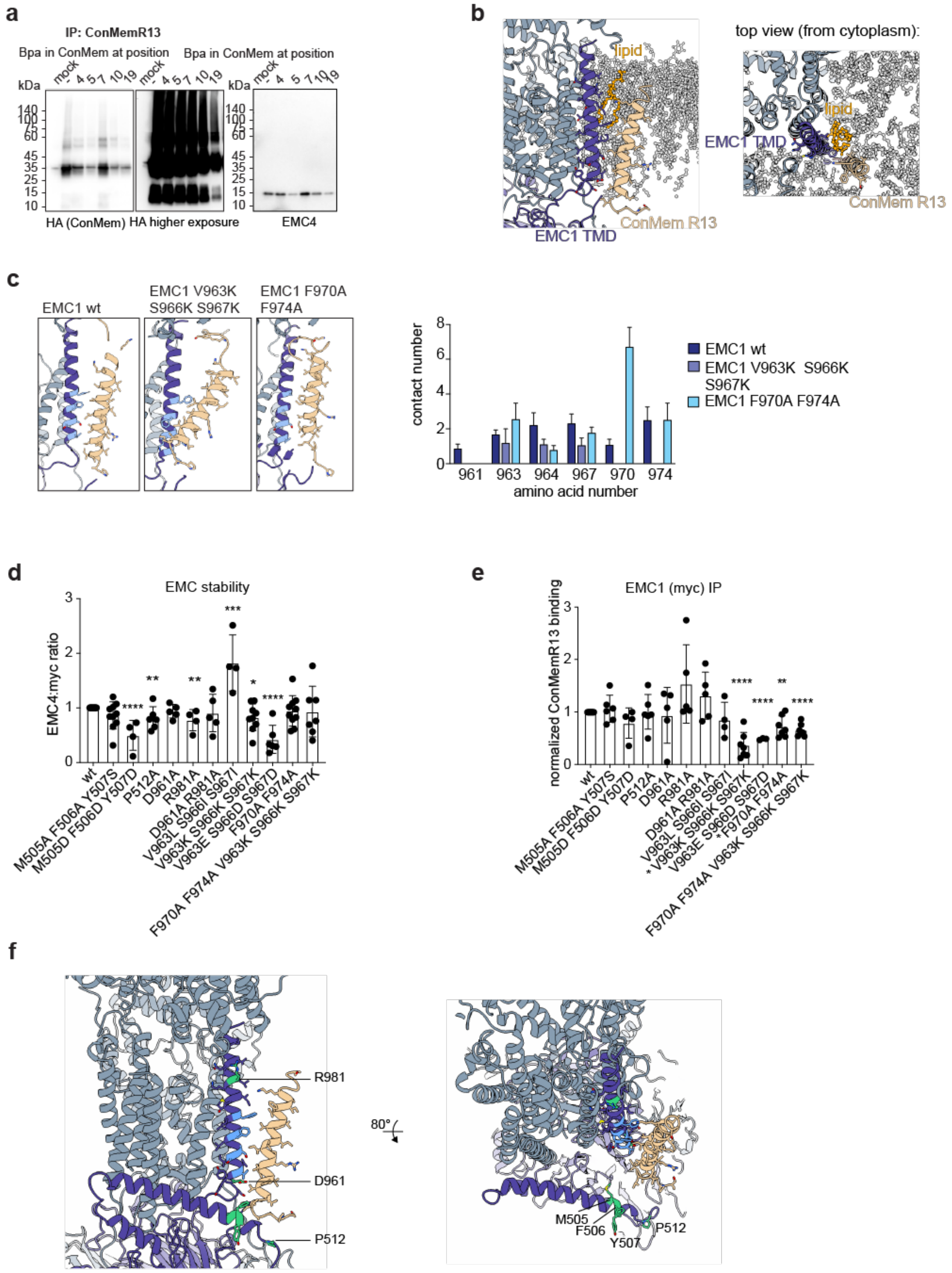

Supplementary Figure 5. Experimental validation of EMC:ConMem 13R binding simulations.

a, Site specific replacement of indicated residues within the transmembrane domain of a strong binding ConMem variant (R13) with Bpa. ConMem and crosslinked species were isolated by immunoprecipitation after UV irradiation. Different exposures of the anti-HA blot

are shown to illustrate the high degree of crosslinking of ConMem R13 in cells. Photocrosslinking does not produce covalent adducts with the integral subunit EMC4.

b, Snapshot of a simulation of EMC and ConMem R13 in an ER-membrane equivalent lipid bilayer shows insertion of a lipid molecule (PE=1-palmitoyl-2-oleoyl phosphatidylethanolamine; highlighted in orange) in the cytoplasmic leaflet between the TMD of EMC1 and ConMem.

c, Mutation of hydrophobic residues in the EMC1 TMD as in Fig. 4 leads to modulated binding *in silico*. Representative poses of ConMem R13 bound to EMC1 wt, EMC1 V963K S966K S967K and EMC1 F970A F974A are shown on the left and the cumulative number of contacts to residues (mean +/- SEM) in the EMC1 TMD throughout the simulations (5 replicates simulated for 100 ns each, data collected every 200 ps) is shown on the right.

d, Effect of EMC1 mutations on overall EMC stability. Myc (EMC1) pulldowns were performed and co-immunoprecipitated endogenous EMC4 was analyzed to assess stability of the EMC complex. Binding was assessed in at least four independent replicates and normalized to EMC1 wt (mean ± SD, **P value < 0.01, ****P value < 0.0001, two-tailed Student's t tests). Mutations shown in Fig. 4c and the ones shown on the left and below were assessed.

e, Mutation of EMC1 residues predicted to interact with ConMem R13 in MD simulations. Effects on ConMem R13 binding were assessed in at least four independent replicates by immunoprecipitation against EMC1-myc and normalized to EMC1-myc wt (mean ± SD, *P value < 0.05, **P value < 0.01, ***P value < 0.001, ****P value < 0.0001, two-tailed Student's t tests). Mutants also depicted in Fig. 4c are marked with an asterisk.

f, Depiction of the EMC1 residues that were mutated in d and e and were shown to not impact ConMem binding or to destabilize the overall EMC (green and labeled). Residues that were shown to affect ConMem R13 binding and are shown in Fig. 4c are colored in blue.

Supplementary Fig. 6

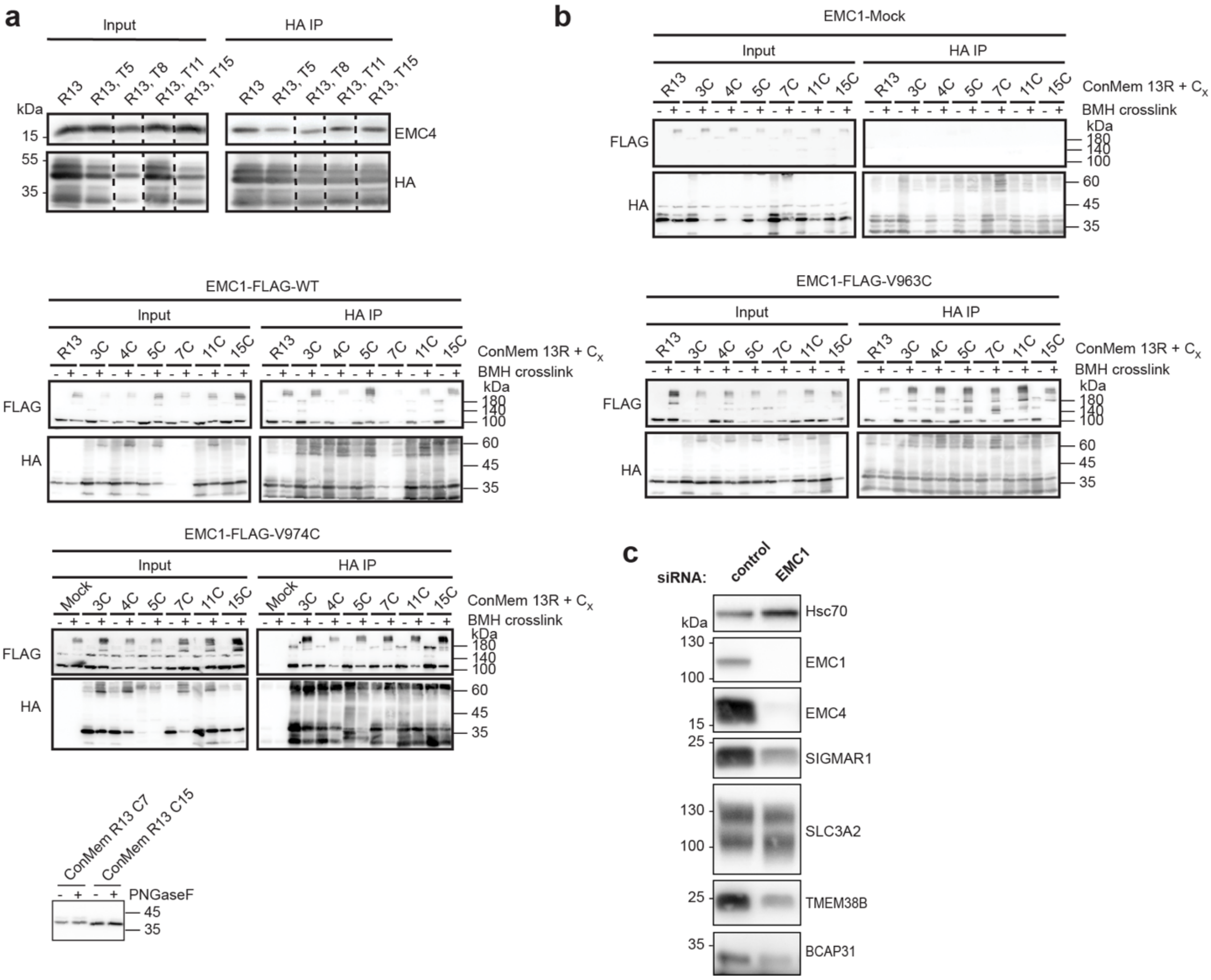

Supplementary Figure 6. Elucidation of essential contact sites in the EMC chaperone-client interaction.

a, Representative western blots of the EMC interaction with ConMem R13 with additional polar amino acids introduced into the N-terminal region of its TMD, assessed via co-immunoprecipitation and used for the quantification depicted in Fig. 4d. The whole cell lysate (input) and HA pulldowns were blotted. Non-relevant lanes were cropped, as indicated by dashed lines.

b, Additional western blots for the assessment of spatial proximity of EMC1 and ConMem residues via BMH crosslinking, supplementing those shown in Fig. 4e. Cells were transfected with ConMem R13 variants containing a cysteine within the transmembrane domain or no cysteine (R13) in addition to EMC1 cysteine mutants. HA blots are included to demonstrate successful ConMem transfection. No crosslinks could be observed upon EMC1 V974C transfection, demonstrating the positional specificity of EMC1:ConMem crosslinks. Furthermore, two representative ConMem R13 cysteine mutants were included in a PNGaseF

digest to demonstrate their correct orientation, using the same principle as depicted in Extended Fig. 2d, top.

c, siRNA-mediated knockdown of EMC1 leads to depletion of clients identified in Fig. 1c at endogenous levels. Western blot of whole cell lysate as respresentative example of quantifications shown in Fig. 4g. Hsc70 serves as loading control

Supplementary Fig. 7

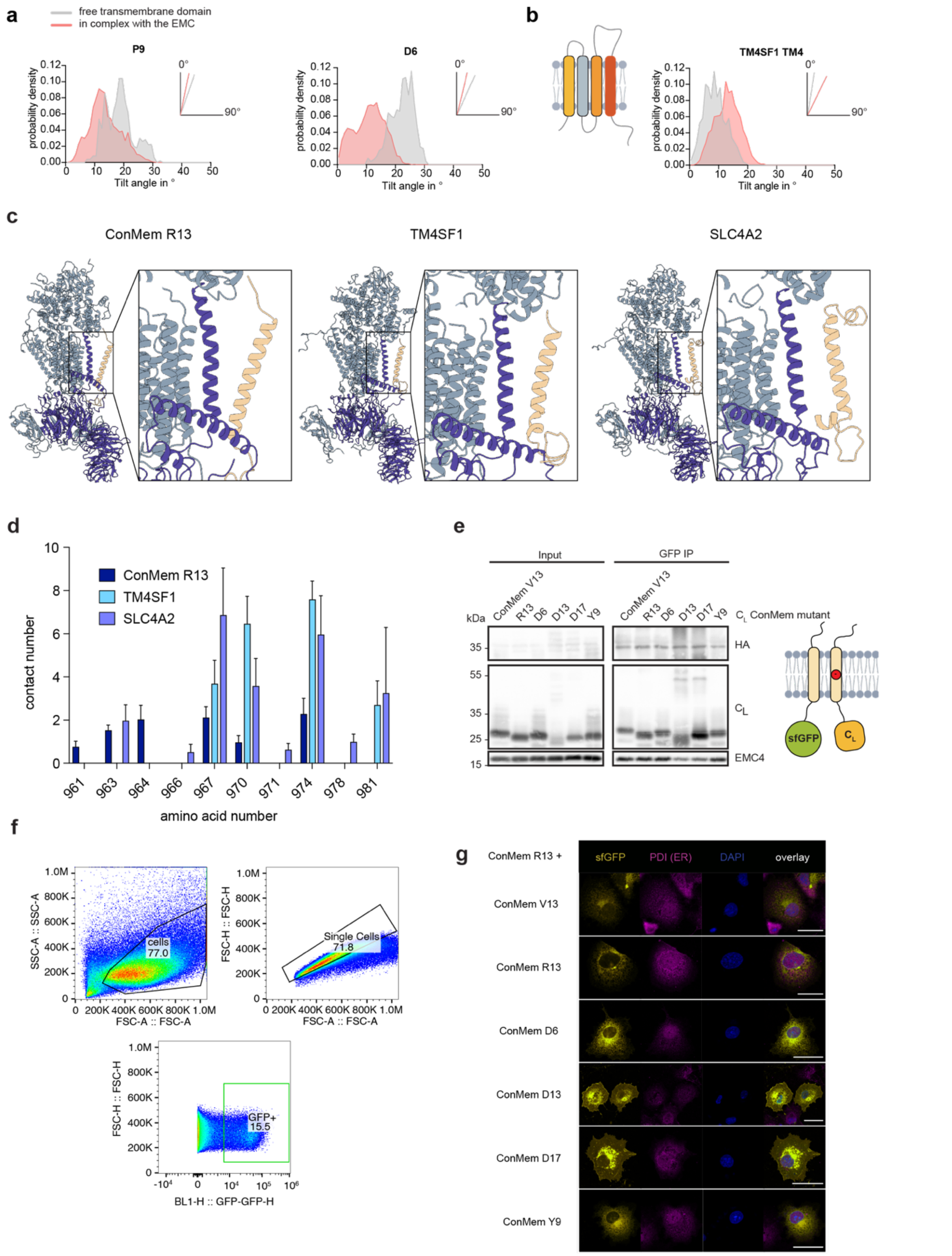

Supplementary Figure 7. Dissecting effects of EMC binding on chaperone clients.

a, Analysis of the TMD tilt towards membrane normal throughout molecular dynamics simulations of selected ConMem variants in isolation (grey) and bound to the EMC1

chaperone site (red). Simulated TMD with a high experimental EMC binding affinity (D6) is tilted to higher angles in isolation, which is significantly reduced upon EMC binding. In contrast, the tilt angle of a weak binder (P9) is not strongly affected by EMC binding (left).

b, Tilt angle analysis as in a, for TMD4 of TMSF4, which was identified in Fig. 3c as a strong EMC binding endogenous transmembrane sequence

c, Snapshots of one representative pose of the MD simulations of the EMC with ConMem R13 (left), TMD4 of TM4SF1 (middle) and TMD3 of SLC4A2 (right). All client TMDs (tan-colored) depict a similar binding pose.

d, Analysis of contacts of the client TMDs with EMC1 during the MD simulations. The natural client TMDs contact the similar residues as ConMem R13, suggesting a similar site of engagement and binding mode.

e, Representative western blots of the ConMem 13R interaction with other ConMem variants and EMC4, assessed *via* co-immunoprecipitation and used for the quantification depicted in Fig. 6b. Both ConMem variants were expressed from a shared promoter using a construct separated by a P2A sequence, one being a ConMem as described previously, and the other having its GFP moiety exchanged to an antibody light chain constant domain (C_L) (see schematic on the right). This allowed to asses interaction by IP-western experiments. The C_L variant contained an arginine at position 13 (red), while different GFP-contanining variants were employed. Binding of the GFP-variant to EMC was assessed in addition to its interaction with the regular GFP-containing ConMem. The whole cell lysate (input) and GFP pulldowns were blotted.

f, Representative dot plots for the gating strategy to identify cells, single cells and GFP positive cells in the flow cytometry assay. The APC:GFP ratio was calculated for the GFP-positive population.

g, Verification of ConMem localization via immunofluorescence micrscoscopy of COS-7 cells to complement the flow cytometry analysis in Figure 6c. For most additionally expressed C_L-ConMem constructs, the R13 ConMem-derived GFP signal can be observed to colocalize with an ER stain. However, when the additionally expressed C_L-ConMems include central residues complementary to R13 (D13 and D17), the GFP-ConMem variant is transported to the cell surface where its GFP signal can be observed. Representative images were chosen that depict the most commonly observed phenotype amongst all observed cells, though a mixed phenotype could be observed in most experiments, both within most cells and across different cells. Cell-surface transport was the predominant phenotype (>80% of observed cells) in D13 and D17 co-expression experiments, while cell-surface transport was observed at <20% in all other experiments. This distributions were estimated from manual counts.

Supplementary Table 1: Predicted ΔGapp values for TMDs used in this study

Transmembrane domain	Sequence	Predicted ΔGapp (kcal/mol)	Membrane integrated according to experi-mental data?
SIGMAR1	AWAALLLAVAAVLTQVVWLWL	-1.866	Yes
SCARB1 TMD1	VMVIMVAGLVACLLGAVGLAG	-0.453	Yes
SCARB1 TMD2	AQYVLLALGCVLLLVPVICQI	-0.655	Yes
BCAP31 TMD1	AVATFLYAEVFVVLLLCIPFI	-1.026	No
BCAP31 TMD2	IVLLVLIVILVVFFTNGYAVL	-2.284	Yes
BCAP31 TMD3	LYIAGFSLLLSFLLRRLVTLI	-0.717	No
SLC3A2	LLLLFWLGWLGMLAGAVVIIV	-1.998	Yes
TMX4	VFFVIATLVFGLFMGLVLVVI	-2.138	Yes
TOR1AIP2	FWSYGPVILVVLVVAVVASSV	-0.371	Yes
GPX8	VFAVLLSIVLCTVTLFLLQLKFL	-2.349	Yes
ConMem R9R13R15R17	PAAILVIVRVVVRFRIRVVVVVFIIK	1.799	No
ConMem	PAAILVIVVVVVVFIIIVVVVVFIIK	-5.061	Yes
ConMem R13	PAAILVIVVVVVRFIIIVVVVVFIIK	-2.92	Yes
ConMem G13	PAAILVIVVVVVGFIIIVVVVVFIIK	-4.368	Yes
ConMem D13	PAAILVIVVVVVDFIIIVVVVVFIIK	-2.728	Yes
ConMem S13	PAAILVIVVVVVSFIIIVVVVVFIIK	-4.098	Yes
ConMem D6	PAAILDIVVVVVVFIIIVVVVVFIIK	-3.936	Yes
ConMem R6	PAAILRIVVVVVVFIIIVVVVVFIIK	-4.771	Yes
ConMem K13	PAAILVIVVVVVKFIIIVVVVVFIIK	-2.661	Yes
ConMem P13	PAAILVIVVVVVPFIIIVVVVVFIIK	-3.619	Yes
ConMem E13	PAAILVIVVVVVEFIIIVVVVVFIIK	-3.199	Yes
ConMem N13	PAAILVIVVVVVNFIIIVVVVVFIIK	-3.317	Yes
poly-L + 8xR	PLLLLLRLRLRLRLRLRLRLRLLLLK	1.146	No
CD9 TMD1	LLFGFNFIFWLAGIAVLAIGL	-1.351	Yes
CD9 TMD2	FYTGVYILIGAGALMMLVGFL	-1.277	No
CD9 TMD3	MLGLFFGFLLVIFAIEIAAAIWGY	-3.387	Yes
CD9 TMD4	IGAVGIGIAVVMIFGMIFSMILCCAI	-2.379	Yes
TM4SF1 TMD1	IGHSLVGLALLCIAANILLYF	-0.789	Yes
TM4SF1 TMD2	FSGIVGGGLLMLLPAFVFIGL	0.086	No
TM4SF1 TMD4	LFSILLALGGIEFILCLIQVI	-0.918	Yes
ConMem R13 C7	PAAILVCVVVVVRFVIIVVVVVFIIK	-2.494	Yes
ConMem R13 C15	PAAILVIVVVVVRFCIIVVVVVFIIK	-2.623	Yes
ConMem poly-L	PLLLLLLLLLLLLLLLLLLLLLLLLK	-10.506	Yes
Predicted weak binder 1	PLLLLILLGLLLCLLLGLLLGLLLLK	-7.432	Yes
Predicted weak binder 2	PLLLLILLGLLLDLLLPLLLGLLLLK	-4.394	Yes

Predicted weak binder 3	PLLLLILLGLLLCLLLPLLLGLLLLK	-6.76	Yes
Predicted moder-ate binder 1	PLLLLELLRLLLLLLLLRLLLMLLLLK	-5.128	Yes
Predicted moder-ate binder 2	PLLLLQLLPLLLRLLLRLLLFLLLLK	-2.927	Yes
Predicted moder-ate binder 3	PLLLLALLMLLLRLLLRLLLHLLLLK	-3.921	Yes
Predicted strong binder 1	PLLLLDLLRLLLRLLLRLLLALLLLK	-2.439	Yes
Predicted strong binder 2	PLLLLDLLRLLLRLLLRLLLMLLLLK	-2.515	Yes
Predicted strong binder 3	PLLLLDLLRLLLRLLLRLLLLLLLLK	-2.927	Yes

Supplementary Table 2: ConMem dataset used to train first iteration of *ipred*^{EMC}

TM Sequence	Binding affinity	Statistical significance
PAAILVIVVVVVVFIIIVVVVVFIIK	1.000	n.a.
PAAILVIVVVVVAFIIIVVVVVFIIK	1.130	n.s.
PAAILVIVVVVVRFIIIVVVVVFIIK	14.512	**
PAAILVIVVVVVNFIIIVVVVVFIIK	1.813	n.s.
PAAILVIVVVVVDFIIIVVVVVFIIK	1.729	n.s.
PAAILVIVVVVVCFIIIVVVVVFIIK	2.072	n.s.
PAAILVIVVVVVQFIIIVVVVVFIIK	1.434	n.s.
PAAILVIVVVVVEFIIIVVVVVFIIK	2.017	*
PAAILVIVVVVVGFIIIVVVVVFIIK	2.684	n.s.
PAAILVIVVVVVHFIIIVVVVVFIIK	2.124	n.s.
PAAILVIVVVVVIFIIIVVVVVFIIK	1.810	n.s.
PAAILVIVVVVVLFIIIVVVVVFIIK	1.894	n.s.
PAAILVIVVVVVKFIIIVVVVVFIIK	5.253	*
PAAILVIVVVVVMFIIIVVVVVFIIK	2.277	n.s.
PAAILVIVVVVVFFIIIVVVVVFIIK	1.207	n.s.
PAAILVIVVVVVPFIIIVVVVVFIIK	1.091	n.s.
PAAILVIVVVVVSFIIIVVVVVFIIK	2.140	*
PAAILVIVVVVVTFIIIVVVVVFIIK	1.672	n.s.
PAAILVIVVVVVWFIIIVVVVVFIIK	2.337	**
PAAILVIVVVVVYFIIIVVVVVFIIK	2.659	****
PAAILRIVVVVVVFIIIVVVVVFIIK	4.302	*
PAAILVIVRVVVVFIIIVVVVVFIIK	15.309	**
PAAILVIVVVVVRFIIIVVVVVFIIK	23.560	***
PAAILVIVVVVVVFIIRVVVVVFIIK	38.009	****
PAAILVIVVVVVVFIIIVVVRVFIIK	3.341	***
PAAILDIVVVVVVFIIIVVVVVFIIK	4.718	**
PAAILVIVDVVVVFIIIVVVVVFIIK	2.653	n.s.
PAAILVIVVVVVDFIIIVVVVVFIIK	1.029	n.s.
PAAILVIVVVVVVFIIDVVVVVFIIK	2.035	n.s.
PAAILVIVVVVVVFIIIVVVDVFIIK	2.523	**
PAAILEIVVVVVVFIIIVVVVVFIIK	1.223	n.s.
PAAILVIVEVVVVFIIIVVVVVFIIK	0.970	n.s.
PAAILVIVVVVVEFIIIVVVVVFIIK	2.054	*
PAAILVIVVVVVVFIIEVVVVVFIIK	1.344	n.s.
PAAILVIVVVVVVFIIIVVVEVFIIK	1.968	**
PAAILGIVVVVVVFIIIVVVVVFIIK	1.547	*
PAAILVIVGVVVVFIIIVVVVVFIIK	1.076	n.s.
PAAILVIVVVVVGFIIIVVVVVFIIK	2.698	**

PAAILVIVVVVVVFIIGVVVVVFIIK	1.366	n.s.
PAAILVIVVVVVVFIIIVVVGVFIIK	0.660	*
PAAILKIVVVVVVFIIIVVVVVFIIK	3.343	**
PAAILVIVKVVVVFIIIVVVVVFIIK	4.436	*
PAAILVIVVVVVKFIIIVVVVVFIIK	6.384	***
PAAILVIVVVVVVFIIKVVVVVFIIK	7.043	*
PAAILVIVVVVVVFIIIVVVKVFIIK	3.782	**
PAAILPIVVVVVVFIIIVVVVVFIIK	0.699	n.s.
PAAILVIVPVVVVFIIIVVVVVFIIK	0.575	*
PAAILVIVVVVVPFIIIVVVVVFIIK	0.903	n.s.
PAAILVIVVVVVVFIIPVVVVVFIIK	0.922	n.s.
PAAILVIVVVVVVFIIIVVVPVFIIK	1.438	*
PAAILSIVVVVVVFIIIVVVVVFIIK	4.235	*
PAAILVIVSVVVVFIIIVVVVVFIIK	0.821	n.s.
PAAILVIVVVVVSFIIIVVVVVFIIK	1.891	n.s.
PAAILVIVVVVVVFIISVVVVVFIIK	3.891	**
PAAILVIVVVVVVFIIIVVVSVFIIK	3.457	**
PAAILYIVVVVVVFIIIVVVVVFIIK	1.646	n.s.
PAAILVIVYVVVVFIIIVVVVVFIIK	6.024	*
PAAILVIVVVVVYFIIIVVVVVFIIK	3.257	*
PAAILVIVVVVVVFIIYVVVVVFIIK	3.952	n.s.
PAAILVIVVVVVVFIIIVVVYVFIIK	1.237	n.s.

Binding affinity denotes the mean from at least three independent co-IP replicates. *P value < 0.05, **P value < 0.01, ***P value < 0.001, ****P value < 0.0001, two-tailed Student’s t tests.

Supplementary Table 3: Position specific features correlating with EMC binding

AAindex feature	Position	Pearson's correlation coefficient	p value (two-sided)
EISD860102	13	0.89	0
FAUJ880109	13	0.8	0
HUTJ700103	13	0.72	2.00E-05
JOND750102	13	-0.73	1.00E-05
RADA880104	13	-0.74	1.00E-05
RADA880107	13	-0.75	1.00E-05
YUTK870102	13	-0.75	1.00E-05
YUTK870103	13	-0.93	0
YUTK870104	13	-0.93	0
ZIMJ680104	13	0.73	2.00E-05
GUYH850105	13	0.74	1.00E-05
JACR890101	13	-0.78	0
TANS770106	6	0.85	0.00725
VELV850101	6	0.89	0.00336
COSI940101	6	0.89	0.00337
CHOP780206	9	-0.87	0.00492
EISD860102	9	0.89	0.0034
FAUJ880109	9	0.91	0.00159
JOND750102	9	-0.87	0.00541
OOBM770102	9	0.85	0.00729
RADA880107	9	-0.85	0.00699
RICJ880107	9	0.93	0.00067
YUTK870101	9	-0.86	0.00563
YUTK870103	9	-0.92	0.0012
YUTK870104	9	-0.92	0.00117
GUYH850105	9	0.89	0.00277
EISD860102	17	0.89	0.0027
FAUJ880109	17	0.92	0.00104
JOND750102	17	-0.93	0.00087
RADA880104	17	-0.87	0.00445
RICJ880107	17	0.93	0.00089
YUTK870101	17	-0.97	8.00E-05
YUTK870102	17	-0.89	0.00298
YUTK870103	17	-0.99	0
YUTK870104	17	-0.99	1.00E-05
GUYH850105	17	0.88	0.00374
PONP800105	21	-0.86	0.00604
RICJ880113	21	0.85	0.00739
SNEP660101	21	0.84	0.00875
COWR900101	21	-0.85	0.00819

Supplementary Table 4: Additional ConMem dataset used to train second iteration of *ipred*^{EMC}

TM Sequence	Binding affinity	Statistical significance
PLLLVILLGLLLCLLLGLLLGLLLLK	1.076	n.a.
PLLLVILLGLLLDLLLPLLLGLLLLK	1.080	n.s.
PLLLVILLGLLLCLLLPLLLGLLLLK	1.185	n.s.
PLLLVELLRLLLLLLLRLLLMLLLLK	2.801	**
PLLLVQLLPLLLRLLLRLLLFLLLLK	3.862	**
PLLLVALLMLLLRLLLRLLLHLLLLK	4.366	****
PLLLVDLLRLLLRLLLRLLLALLLLK	18.110	****
PLLLVDLLRLLLRLLLRLLLMLLLLK	13.702	***
PLLLVDLLRLLLRLLLRLLLLLLLLK	22.232	****
LLFGFNFIFWLAGIAVLAIGL	5.983	****
LFSILLALGGIEFILCLIQVI	3.676	****
SNHLEYLVGRVWIGFWLVFLALLMVALEGSFLV	8.234	***
PRAILVIVVVVVRFIIIVVVVVFIIK	5.253	****
PYAILVIVVVVVRFIIIVVVVVFIIK	2.277	****
PDAILVIVVVVVRFIIIVVVVVFIIK	1.207	**
PAASLVSVVVSVRFIIIVVVVVFIIK	9.089	**
PAATIVIVVVIVRFIIIVVVVVFIIK	15.504	***
PACSLTCCVVTCRFIIIVVVVVFIIK	8.602	**

Binding affinity denotes the mean from at least three independent co-IP replicates. **P value < 0.01, ***P value < 0.001, ****P value < 0.0001, two-tailed Student's t tests.

Supplementary Table 5: Protein sequences of EMC subunits used for the Alphafold 2 model

EMC subunit	Uniprot Code		Sequence
			1 10 20 30 40 50 60 70
EMC1	Q8N766	1	YEDQVGKFDWRQQYVGKVKFASLEFSPGSKKLVVATEKNVIAALNSRT
		71	GEILWRHVDKGTAEGAVDAMLLHGQDVITVSNGGRIMRSWETNIGGLNWEITLDSGSFQALGLVGLQESV
		141	RYIAVLKKTTLALHHLSSGHLKWVEHLPESDSIHYQMVYSYGSGVVWALGVVPFSHVNIVKFNVEDGEIV
		211	QQVRVSTPWLQHLSGACGVVDEAVLVCPDPSSRSLQTLALETEWELRQIPLQSLDLEFGSGFQPRVLPTQ
		281	PNPVDASRAQFFLHLSPSHYALLQYHYGTLSLLKNFPQTALVSFATTGEKTVAAVMACRNEVQKSSSSED
		351	GSMGSFSEKSSSKDSLACFNQTYTINLYLVETGRRLLDTTITFSLEQSGTRPERLYIQVFLKKDDSVGYR
		421	ALVQTEDHLLLFLQQLAGKVVLWSREESLAEVVCLEMVDLPLTGAQAELEGEFGKKADGLLGMFLKRLSS
		491	QLILLQAWTSHLWKMFYDARKPRSQIKNEINIDTLARDEFNLQKMMVMVTASGKLFGIESSSGTILWKQY
		561	LPNVKPDSSFKLMVQRTTAHFPHPPQCTLLVKDKESGMSSLYVFNPIFGKWSQVAPPVLKRPILQSLLLP
		631	VMDQDYAKVLLLIDDEYKVTAFPATRNVLRQLHELAPSIFFYLVDAEQGRLCGYRLRKDLTTELSWELTI
		701	PPEVQRIVKVKGKRSSEHVHSQGRVMGDRSVLYKSLNPNLLAVVTESTDAHHERTFIGIFLIDGVTGRII
		771	HSSVQKKAKGPVHIVHSENWVVYQYWNTKARRNEFTVLELYEGTEQYNATAFSSLDRPQLPQVLQQSYIF
		841	PSSISAMEATITERGITSRHLLIGLPSGAILSLPKALLDPRRPEIPTEQSREENLIPYSPDVQIHAERFI
		911	NYNQTVSRMRGIYTAPSGLESTCLVVAYGLDIYQTRVYPSKQFDVLKDDYDYVLISSVLFGLVFATMITK
		981	RLAQVKLLNRAWR
			1 10 20 30 40 50 60 70
EMC2	Q15006	1	AKVSELYDVTWEEMRDKMRKWREENSRNSEQIVEVGEELINEYASKLGDDIWIIYEQVMIAALDYGRDD
		71	LALFCLQELRRQFPGSHRVKRLTGMRFEAMERYDDAIQLYDRILQEDPTNTAARKRKIAIRKAQGKNVEA
		141	IRELNEYLEQFVGDQEAWHELAELYINEHDYAKAAFCLEELMMTNPHNHLYCQQYAEVKYTQGGLENLEL
		211	SRKYFAQALKLNNRNMRALFGLYMSASHIASNPKASAKTKKDNMKYASWAASQINRAYQFAGRSKKETKY
		281	SLKAVEDMLETLQITQS
			1 10 20 30 40 50 60 70
EMC3	Q9P0I2	1	AGPELLLDSNIRLWVVLPIVIITFFVGMIRHYVSILLQSDKKLTQEQVSDSQVLIRSRVLRENGKYIPK
		71	QSFLTRKYYFNNPEDGFFKKTKRKVVPPSPMTDPTMLTDMMKGNVTNVLPMILIGGWINMTFSGFVTTKV
		141	PFPLTLRFKPMLQQGIELLTLDASWVSSASWYFLNVFGLRSIYSLILGQDNAADQSRMMQEQMTGAAMAM
		211	PADTNKAFKTEWEALELTDHQWALDDVEEELMAKDLHFEGMFKKELQTSIF
			1 10 20 30 40 50 60 70
EMC4	Q5J8M3	1	TAQGGLVANRGRRFKWAIELSGPGGGSRGRSDRGSGQGDSLYPVGYLDKQVPDTSVQETDRILVEKRCW
		71	DIALGPLKQIPMNLFIMYMAGNTISIFPTMMVCMMAWRPIQALMAISATFKMLESSSQKFLQGLVYLIGN
		141	LMGLALAVYKCQSMGLLPTHASDWLAFIEPPERMEFSGGGLLL
			1 10 20 30 40 50 60 70
EMC5	6Z3W*	1	MAPSLWKGLVGIGLFALAHAAFSAAQHRSYMRLTEKEDESLPIDIVLQTLLAFAVTCYGIVHIAGEFKDM
		71	DATSELKNKTFDTLRNHPSFYVFNHRGRVLFRPSDTANSSNQDALSSNTSLKLRKLESLRR
			1 10 20 30 40 50 60 70
EMC6	Q9BV81	1	AAVVAKREGPPFISEAAVRGNAAVLDYCRTSVSALSGATAGILGLTGLYGFIFYLLASVLLSLLLILKA
		71	GRRWNKYFKSRRPLFTGGLIGGLFTYVLFWTFLYGMVHVY
			1 10 20 30 40 50 60 70
EMC7	Q9NPA0	1	SEVPGAAAEGSGGSGVGIGDRFKIEGRAVVPGVKPQDWISAARVLVD
		71	GEEHVGFLKTDGSFVVHDIPSGSYVVEVVSPAYRFDPVRVDITSKGKMRARYVNYIKTSEVVRLPYPLQM
		141	KSSGPPSYFIKRESWGWTDFLMNPMVMMMVLPLLIFVLLPKVVNTSDPDMRREMEQSMNMLNSNHELPDV
		211	SEFMTRLFSSKSSGKSSSGSSKTGKSGAGKRR
			1 10 20 30 40 50 60 70
EMC9	Q9Y3B6	1	MGEVEISALAYVKMCLHAARYPHAAVNGLFLAPAPRSGECLCLTDCVPLFHSHLALSVMLEVALNQVDVWGAQAGL
		71	VVAGYYHANAAVNDQSPGPLALKIAGRIAEFFPDAVLIMLDNQKLVPQPRVPPVIVLENQGLRWVPKDKNLVMWRD
		141	WEESRQMVGALLEDRAHQHLVDFDCHLDDIRQDWTNQRLNTQITQWVGPTNGNGNA
			1 10 20 30 40 50 60 70
EMC10	Q5UCC4	1	RGSGCRAGTGARGAGAEGREGEACGTVGLLLEHSFEIDDSANFRK
		71	RGSLLWNQQDGTLSLSQRQLSEEERGRLRDVAALNGLYRVRIPRRPGALDGLEAGGYVSSFVPACSLVES
		141	HLSDQLTLHVDVAGNVVGVSVVTHPGGCRGHEVEDVDLELFNTSVQLQPPTTAPGPETAAFIERLEMEQA
		211	QKAKNPQEQKSFFAKYWMYIIPVVLFLMMSGAPDTGGQGGGGGGGGGGGSGR

SupplementaryTable 6: Number of membrane components in the upper and lower ER membrane leaflet

Component		Upper Leaflet	Lower Leaflet
Cholesterol		32	28
Sphingomyeline	d18:1/16:0	12	12
Cardiolipin (-1)	18:1/18:1/18:1/18:1	4	4
Phospholipids			
PC	16:0/16:1	40	40
	16:0/18:0	16	16
	16:0/18:1	104	104
	16:0/18:2	24	24
	18:1/18:1	36	36
PE	16:0/18:1	32	32
	16:0/18:2	4	4
	18:0/18:1	16	16
	18:1/18:1	32	32
PS	18:0/18:1	8	8
	18:0/18:2	4	4
PI	16:0/18:1	8	8
	18:0/18:2	12	12
	18:0/20:4	20	20

Supplementary References

1 Bloemeke, N. *et al.* Intramembrane client recognition potentiates the chaperone functions of calnexin. *Embo j* **41**, e110959 (2022). https://doi.org:10.15252/embj.2022110959

2 Hessa, T. *et al.* Molecular code for transmembrane-helix recognition by the Sec61 translocon. *Nature* **450**, 1026-1030 (2007). https://doi.org:nature06387 [pii] 10.1038/nature06387
