# Supplementary material for: The EMC acts as a chaperone for membrane proteins
Source: Nat Commun. 2025 Aug 2;16:7097. doi: 10.1038/s41467-025-62109-x (PMC12317982; doi:10.1038/s41467-025-62109-x)
Supplement: Supplementary file 2 — Description of Additional Supplementary Files [file 41467_2025_62109_MOESM2_ESM.pdf]

Description of Additional Supplementary Files

File name: Supplementary Data 1
Description: Significantly enriched MS hits.

File name: Supplementary Data 2
Description: EMC clients reported in the literature.
