# Supplementary material for: The EMC acts as a chaperone for membrane proteins
Source: Nat Commun. 2025 Aug 2;16:7097. doi: 10.1038/s41467-025-62109-x (PMC12317982; doi:10.1038/s41467-025-62109-x)
Supplement: Supplementary file 5 — Reporting summary [file 41467_2025_62109_MOESM5_ESM.pdf]

nature portfolio

Corresponding author(s): Matthias Feige

Last updated by author(s): Jun 18, 2025

Reporting Summary

Nature Portfolio wishes to improve the reproducibility of the work that we publish. This form provides structure for consistency and transparency in reporting. For further information on Nature Portfolio policies, see our Editorial Policies and the Editorial Policy Checklist.

Statistics

For all statistical analyses, confirm that the following items are present in the figure legend, table legend, main text, or Methods section.

n/a	Confirmed	
☐	☒	The exact sample size (*n*) for each experimental group/condition, given as a discrete number and unit of measurement
☐	☒	A statement on whether measurements were taken from distinct samples or whether the same sample was measured repeatedly
☐	☒	The statistical test(s) used AND whether they are one- or two-sided<br>*Only common tests should be described solely by name; describe more complex techniques in the Methods section.*
☒	☐	A description of all covariates tested
☐	☒	A description of any assumptions or corrections, such as tests of normality and adjustment for multiple comparisons
☐	☒	A full description of the statistical parameters including central tendency (e.g. means) or other basic estimates (e.g. regression coefficient) AND variation (e.g. standard deviation) or associated estimates of uncertainty (e.g. confidence intervals)
☐	☒	For null hypothesis testing, the test statistic (e.g. *F*, *t*, *r*) with confidence intervals, effect sizes, degrees of freedom and *P* value noted<br>*Give P values as exact values whenever suitable.*
☒	☐	For Bayesian analysis, information on the choice of priors and Markov chain Monte Carlo settings
☒	☐	For hierarchical and complex designs, identification of the appropriate level for tests and full reporting of outcomes
☐	☒	Estimates of effect sizes (e.g. Cohen's *d*, Pearson's *r*), indicating how they were calculated

Our web collection on statistics for biologists contains articles on many of the points above.

Software and code

Policy information about availability of computer code

Data collection	LAS X (Leica), timsControl (Bruker), Attune Cytometric Software (Thermo Fisher), NAMD 3.0b3
Data analysis	ImageJ2 Fiji version 2.14.0/1.54f, Spectronaut 18.0 (Biognosys), Perseus 1.6.2.3, FlowJo 10 (BD Life Sciences), UCSF ChimeraX 1.6.1, Bio-1D 15.08 (Vilber Lourmat), Prism 10 (GraphPad), VMD 2.14, Python 3.8, Adobe Illustrator 2025

For manuscripts utilizing custom algorithms or software that are central to the research but not yet described in published literature, software must be made available to editors and reviewers. We strongly encourage code deposition in a community repository (e.g. GitHub). See the Nature Portfolio guidelines for submitting code & software for further information.

Data

Policy information about availability of data

All manuscripts must include a data availability statement. This statement should provide the following information, where applicable:

- Accession codes, unique identifiers, or web links for publicly available datasets
- A description of any restrictions on data availability
- For clinical datasets or third party data, please ensure that the statement adheres to our policy

Further information and requests for resources and reagents should be directed to and will be fulfilled by the lead contact Matthias J Feige (matthias.feige@tum.de). All primary data of this study will be shared upon request from the lead contact.
The code for the machine learning algorithm will be published on Github upon acceptance.

Any additional information required to reanalyze the data reported in this work is available from the lead contact upon request.

Research involving human participants, their data, or biological material

Policy information about studies with human participants or human data. See also policy information about sex, gender (identity/presentation), and sexual orientation and race, ethnicity and racism.

Reporting on sex and gender	N/A
Reporting on race, ethnicity, or other socially relevant groupings	N/A
Population characteristics	N/A
Recruitment	N/A
Ethics oversight	N/A

Note that full information on the approval of the study protocol must also be provided in the manuscript.

Field-specific reporting

Please select the one below that is the best fit for your research. If you are not sure, read the appropriate sections before making your selection.

☒ Life sciences ☐ Behavioural & social sciences ☐ Ecological, evolutionary & environmental sciences

For a reference copy of the document with all sections, see nature.com/documents/nr-reporting-summary-flat.pdf

Life sciences study design

All studies must disclose on these points even when the disclosure is negative.

Sample size	No sample size calculations were performed. Biochemical experiments and flow cytometry were performed in true replicates (different passages of cells, different days) to ensure reproducibility. All biochemical experiments were repeated at least three times to allow for statistical evaluation. Flow cytometry experiments were repeated in five independent replicates and at least 10,000 cells expressing the reporter were analyzed per experiment.
Data exclusions	For western blot quantifications, extreme outliers were identified using Prism's inbuilt ROUT method (Robust regression and Outlier removal) with a Q coefficient of 10 %. Outliers were excluded from further analysis
Replication	Reproducibility and reliability of the data has been ensured in multiple ways. All biochemical experiments have been performed at least in independent triplicates on different occasions yielding the same result.
Randomization	Sample randomization is not applicable to our biochemical and cell biological assays and hence was not performed.
Blinding	No blinding was performed.

Reporting for specific materials, systems and methods

We require information from authors about some types of materials, experimental systems and methods used in many studies. Here, indicate whether each material, system or method listed is relevant to your study. If you are not sure if a list item applies to your research, read the appropriate section before selecting a response.

Materials & experimental systems

n/a	Involved in the study	
☐	☒	Antibodies
☐	☒	Eukaryotic cell lines
☒	☐	Palaeontology and archaeology
☒	☐	Animals and other organisms
☒	☐	Clinical data
☒	☐	Dual use research of concern
☒	☐	Plants

Methods

n/a	Involved in the study	
☒	☐	ChIP-seq
☐	☒	Flow cytometry
☒	☐	MRI-based neuroimaging

Antibodies

Antibodies used	EMC4 (Abcam, ab184544), EMC1 (Novus Biologicals, NBP2-59097 and NBP3-18427), HA.11 (Biolegend, Poly9023), myc clone 4A6 upstate (Sigma-Aldrich, 05-724), FLAG (Sigma-Aldrich, F7425), NWSHPQFEK 5A9F9 (Genscript, A01732), lambda-UNLB (Southern Biotech, SBA-1060-01), TMEM38B (Proteintech, 19919-1-AP), Sigmar1 (Proteintech, 15168-1-AP), Slc3A2 (Proteintech, 15193-1-AP), BCAP31 (Proteintech, 11200-1-AP), Hsc70 (Santa Cruz Biotechnology, sc-7298), GAPDH-HRP (Proteintech, HRP-60004), Calnexin (Santa Cruz Biotechnology, sc-23954), APC-coupled anti-GFP antibody (Biolegend, 338010), TexasRed-coupled anti-mouse IgG (Thermo Fisher, PA1-28626), Alexa647-coupled anti-PDI (Santa Cruz Biotechnology, sc-74551), ILVBL (Proteintech, 11220-1-AP), PERK (Proteintech, 20582-1-AP), EMC2 (Proteintech, 25443-1-AP), Slc7A5 (Proteintech, 28670-1-AP), anti-rabbit IgG-HRP (Santa Cruz Biotechnology, sc-2357)
Validation	EMC4: validation by siRNA knockdown (not shown), widely used in the field e.g. Miller-Vedam et al. 2020, Tian et al. 2019, Coelho et al. 2019<br>EMC1: validation by siRNA knockdown (shown) and overexpression (shown) of the target protein and analysis on a western blot<br>HA.11: validation on supplier website, in different other studies and by overexpression of HA-tagged proteins vs. untagged<br>myc clone 4A6: validation on supplier website, in different other studies and by overexpression of myc-tagged proteins vs. untagged<br>FLAG: validation on supplier website, in different other studies and by overexpression of FLAG-tagged proteins vs. untagged<br>NWSHPQFEK 5A9F9: validation on supplier website, in different other studies and by overexpression of twinStrep-tagged proteins vs. untagged<br>lambda-UNLB: validation on supplier website, in different other studies and by overexpression of CL-tagged proteins vs. untagged<br>TMEM38B: validation on supplier website and by overexpression of the target protein and analysis on a western blot (not shown)<br>SLC3A2: validation on supplier website and by overexpression of the target protein and analysis on a western blot (not shown)<br>Sigmar1: validation on supplier website and by overexpression of the target protein and analysis on a western blot (not shown)<br>BCAP31: validation on supplier website and by overexpression of the target protein and analysis on a western blot (not shown)<br>Hsc70: validation on supplier website<br>GAPDH-HRP: validation on supplier website<br>Calnexin: validation in Calnexin knockout cells (not shown) and on supplier website<br>APC-coupled anti-GFP antibody: validation on supplier website, each experiment included a negative control (not expressing GFP) which was not recognized by the antibody (not shown)<br>TexasRed-coupled anti-mouse IgG: validation on supplier website<br>Alexa647-coupled anti-PDI: validation on supplier website<br>ILVBL: validation on supplier website<br>PERK: validation on supplier website<br>EMC2: validation on supplier website and by EMC knockdown (not shown)<br>Slc7A5: validation on supplier website<br>HRP-coupled anti-rabbit IgG: validation on supplier website

Eukaryotic cell lines

Policy information about cell lines and Sex and Gender in Research

Cell line source(s)	293T cells (Sigma-Aldrich, 12022001, human, female)<br>Cos7 cells (Sigma-Aldrich 87021302, african green monkey, male)
Authentication	cells were obtained from Sigma-Aldrich and not further authenticated
Mycoplasma contamination	all cells were regularly tested negative for mycoplasma contamination
Commonly misidentified lines (See ICLAC register)	None used.

Plants

Seed stocks	*Report on the source of all seed stocks or other plant material used. If applicable, state the seed stock centre and catalogue number. If plant specimens were collected from the field, describe the collection location, date and sampling procedures.*
Novel plant genotypes	*Describe the methods by which all novel plant genotypes were produced. This includes those generated by transgenic approaches, gene editing, chemical/radiation-based mutagenesis and hybridization. For transgenic lines, describe the transformation method, the number of independent lines analyzed and the generation upon which experiments were performed. For gene-edited lines, describe the editor used, the endogenous sequence targeted for editing, the targeting guide RNA sequence (if applicable) and how the editor was applied.*
Authentication	*Describe any authentication procedures for each seed stock used or novel genotype generated. Describe any experiments used to assess the effect of a mutation and, where applicable, how potential secondary effects (e.g. second site T-DNA insertions, mosiacism, off-target gene editing) were examined.*

Flow Cytometry

Plots

Confirm that:

☒ The axis labels state the marker and fluorochrome used (e.g. CD4-FITC).

☒ The axis scales are clearly visible. Include numbers along axes only for bottom left plot of group (a 'group' is an analysis of identical markers).

☒ All plots are contour plots with outliers or pseudocolor plots.

☒ A numerical value for number of cells or percentage (with statistics) is provided.

Methodology

Sample preparation	293T cells were transiently transfected with constructs encoding for GFP-ConMem R13 and a CL-ConMem variant separated by a P2A peptide skipping sequence. 24 hours after transfection, cells were washed with PBS and gently dissociated from the growth plate and incubated for 30 min with an APC-coupled anti_GFP antibody (Biolegend, 338010) in the dark. Excess antibody was removed in two subsequent washing steps with PBS before cells were directly analyzed by flow cytometry.
Instrument	Attune NxT flow cytometer (ThermoFisher)
Software	FlowJo 10 (BD Life Sciences)
Cell population abundance	GFP positive populations ranged from 5 to 30 % of the immediate parental population (single cells).
Gating strategy	Gating was performed based on forward and side scatter to select intact cells and omit fragments. Doublet discrimination was performed based on the area to height ratio observed in the forward scatter signal. GFP gating was performed in a GFP-H vs. FSC-H dot plot showing a distinct separation between the untransfected negative population and GFP-fluorescent cells. All gates were drawn stringently, excluding intermediate populations.

☒ Tick this box to confirm that a figure exemplifying the gating strategy is provided in the Supplementary Information.
