# Supplementary figures and images for: The EMC acts as a chaperone for membrane proteins
Source: Nat Commun. 2025 Aug 2;16:7097. doi: 10.1038/s41467-025-62109-x (PMC12317982; doi:10.1038/s41467-025-62109-x)

Figure 1b

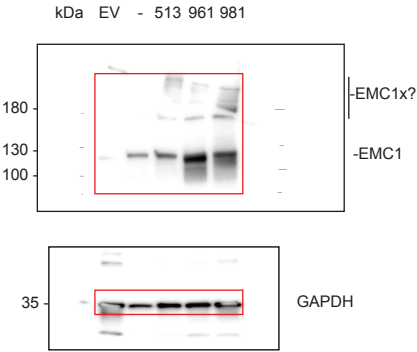

Figure 4a

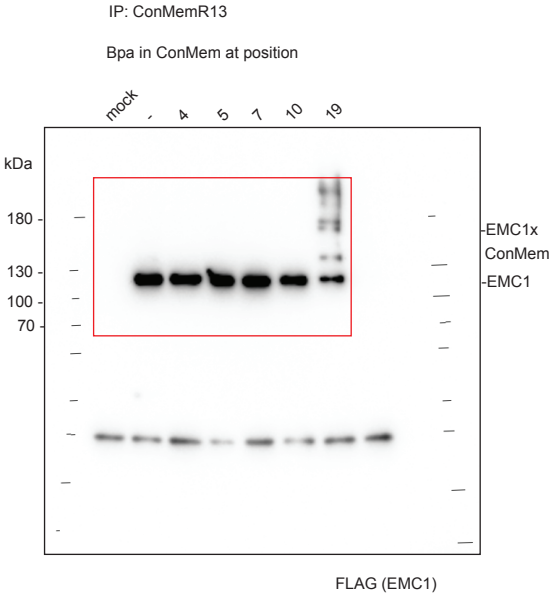

Figure 4e

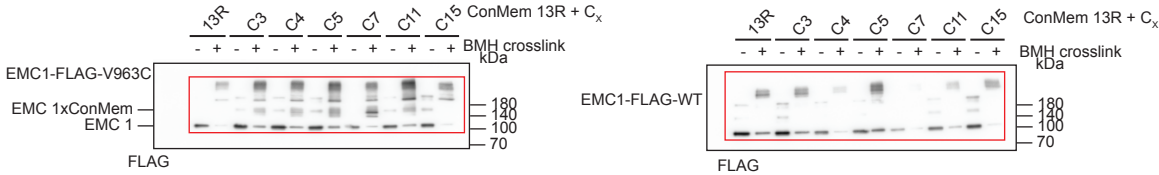

Figure 6d - left

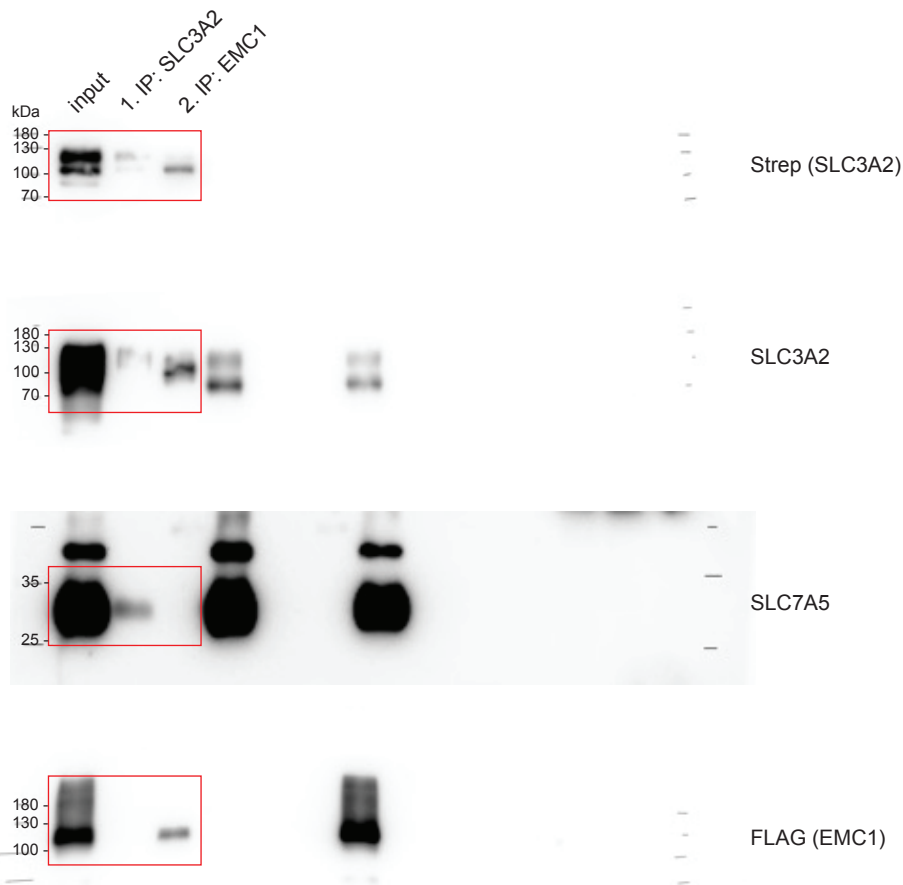

Figure 6d - right

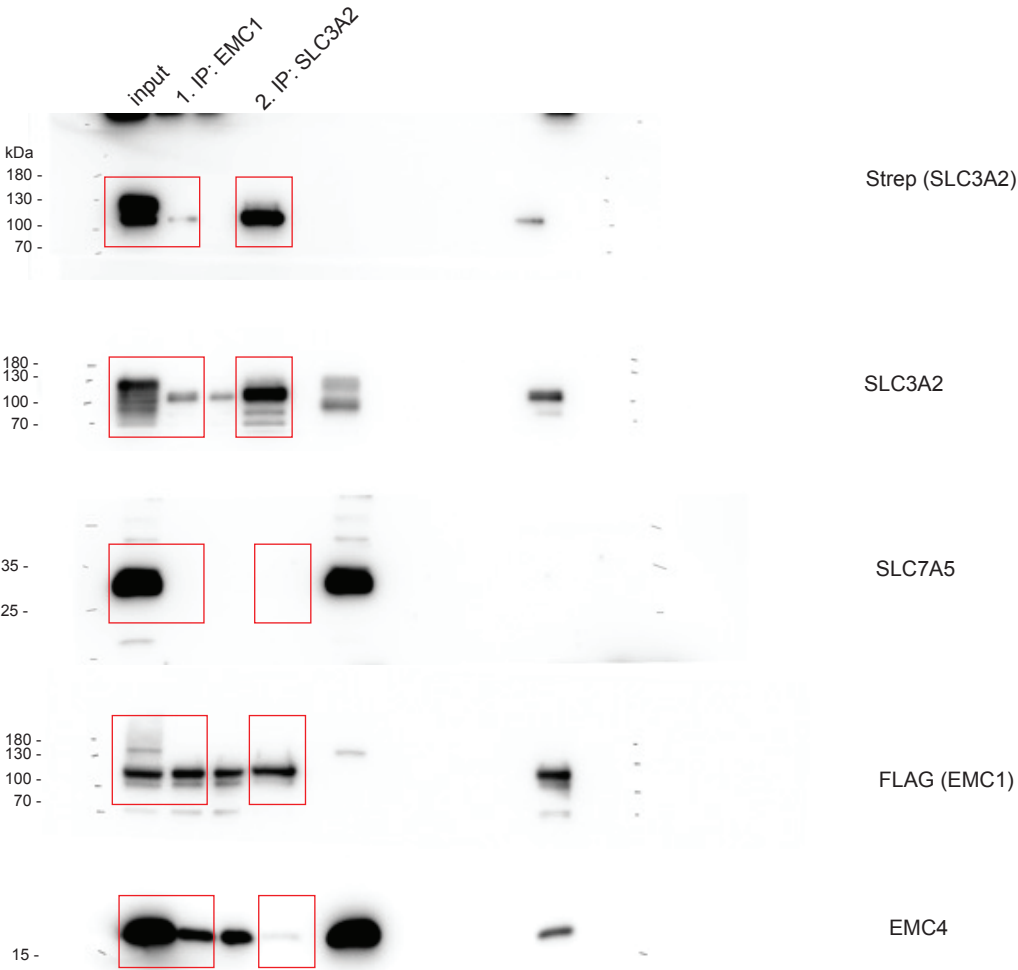

Supplement: Supplementary file 9 — Source Data [file 41467_2025_62109_MOESM9_ESM.zip › sourcedata.pdf]
